# Supplementary material for: Causal Relationship between Aging and Anorexia Nervosa: A White-Matter-Microstructure-Mediated Mendelian Randomization Analysis
Source: Biomedicines. 2024 Aug 16;12(8):1874. doi: 10.3390/biomedicines12081874 (PMC11351342; doi:10.3390/biomedicines12081874)
Supplement: Supplementary file 1 [file biomedicines-12-01874-s001.zip › Supplementary Figure/Figure S4/Singe SNP plot of MR analysis for TL to ubm-b-1565.pdf]

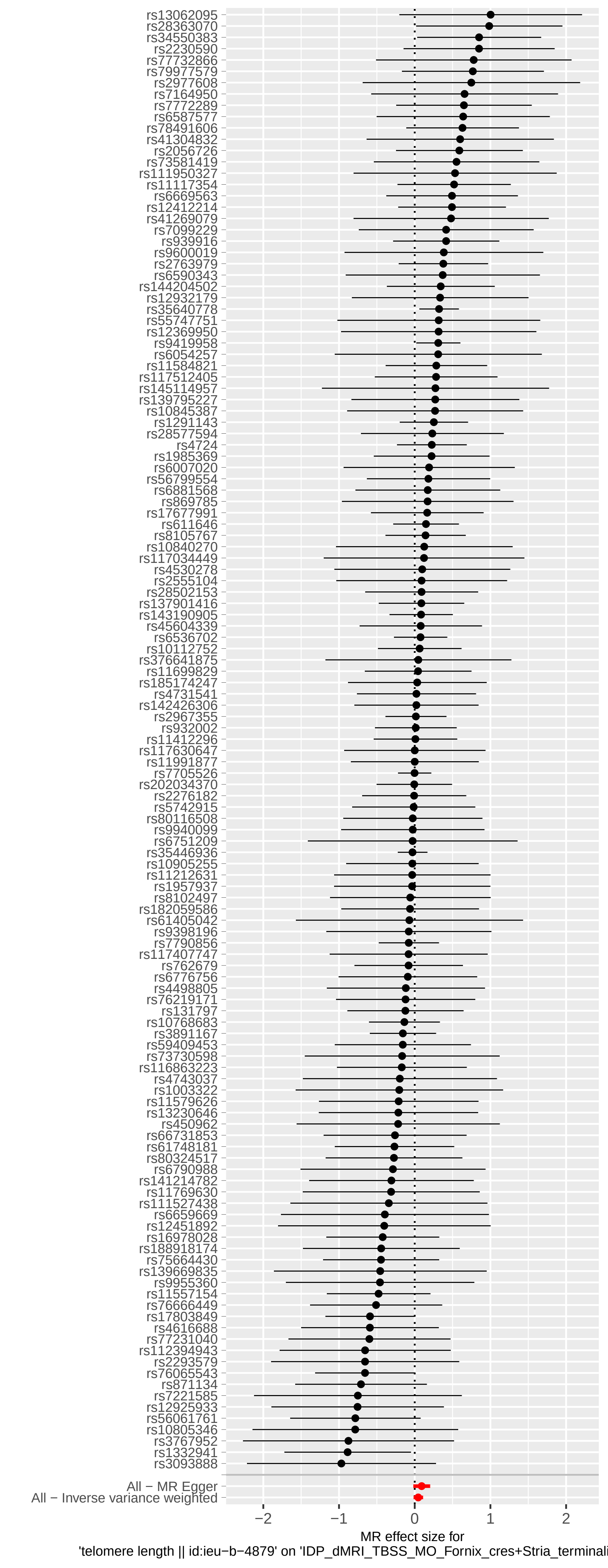

rs13062095
rs28363070
rs34550383
rs2230590
rs77732866
rs79977579
rs2977608
rs7164950
rs7772289
rs6587577
rs78491606
rs41304832
rs2056726
rs73581419
rs111950327
rs11117354
rs6669563
rs12412214
rs41269079
rs7099229
rs939916
rs9600019
rs2763979
rs6590343
rs144204502
rs12932179
rs35640778
rs55747751
rs12369950
rs9419958
rs6054257
rs11584821
rs117512405
rs145114957
rs139795227
rs10845387
rs1291143
rs28577594
rs4724
rs1985369
rs6007020
rs56799554
rs6881568
rs869785
rs17677991
rs611646
rs8105767
rs10840270
rs117034449
rs4530278
rs2555104
rs28502153
rs137901416
rs143190905
rs45604339
rs6536702
rs10112752
rs376641875
rs11699829
rs185174247
rs4731541
rs142426306
rs2967355
rs932002
rs11412296
rs117630647
rs11991877
rs7705526
rs202034370
rs2276182
rs5742915
rs80116508
rs9940099
rs6751209
rs35446936
rs10905255
rs11212631
rs1957937
rs8102497
rs182059586
rs61405042
rs9398196
rs7790856
rs117407747
rs762679
rs6776756
rs4498805
rs76219171
rs131797
rs10768683
rs3891167
rs59409453
rs73730598
rs116863223
rs4743037
rs1003322
rs11579626
rs13230646
rs450962
rs66731853
rs61748181
rs80324517
rs6790988
rs141214782
rs11769630
rs111527438
rs6659669
rs12451892
rs16978028
rs188918174
rs75664430
rs139669835
rs9955360
rs11557154
rs76666449
rs17803849
rs4616688
rs77231040
rs112394943
rs2293579
rs76065543
rs871134
rs7221585
rs12925933
rs56061761
rs10805346
rs3767952
rs1332941
rs3093888
All − MR Egger
All − Inverse variance weighted
−2
−1
0
1
2
MR effect size for
'telomere length || id:ieu−b−4879' on 'IDP_dMRI_TBSS_MO_Fornix_cres+Stria_terminali
